# Alkylation induced cerebellar degeneration dependent on Aag and Parp1 does not occur via previously established cell death mechanisms

**DOI:** 10.1371/journal.pone.0184619

**Published:** 2017-09-08

**Authors:** Carrie M. Margulies, Isaac Alexander Chaim, Aprotim Mazumder, June Criscione, Leona D. Samson

**Affiliations:** 1 Department of Biological Engineering, Massachusetts Institute of Technology, Cambridge, Massachusetts, United States of America; 2 Center for Environmental Health Sciences, Massachusetts Institute of Technology, Cambridge, Massachusetts, United States of America; 3 Department of Biology, Massachusetts Institute of Technology, Cambridge, Massachusetts, United States of America; 4 David H. Koch Institute for Integrative Cancer Research, Massachusetts Institute of Technology, Cambridge, Massachusetts, United States of America; University of South Alabama Mitchell Cancer Institute, UNITED STATES

## Abstract

Alkylating agents are ubiquitous in our internal and external environments, causing DNA damage that contributes to mutations and cell death that can result in aging, tissue degeneration and cancer. Repair of methylated DNA bases occurs primarily through the base excision repair (BER) pathway, a multi-enzyme pathway initiated by the alkyladenine DNA glycosylase (Aag, also known as Mpg). Previous work demonstrated that mice treated with the alkylating agent methyl methanesulfonate (MMS) undergo cerebellar degeneration in an Aag-dependent manner, whereby increased BER initiation by Aag causes increased tissue damage that is dependent on activation of poly (ADP-ribose) polymerase 1 (Parp1). Here, we dissect the molecular mechanism of cerebellar granule neuron (CGN) sensitivity to MMS using primary *ex vivo* neuronal cultures. We first established a high-throughput fluorescent imaging method to assess primary neuron sensitivity to treatment with DNA damaging agents. Next, we verified that the alkylation sensitivity of CGNs is an intrinsic phenotype that accurately recapitulates the *in vivo* dependency of alkylation-induced CGN cell death on Aag and Parp1 activity. Finally, we show that MMS-induced CGN toxicity is independent of all the cellular events that have previously been associated with Parp-mediated toxicity, including mitochondrial depolarization, AIF translocation, calcium fluxes, and NAD^+^ consumption. We therefore believe that further investigation is needed to adequately describe all varieties of Parp-mediated cell death.

## Introduction

DNA alkylation damage, left unrepaired, is mutagenic and cytotoxic, ultimately contributing to aging, tissue degeneration and cancer. Several different DNA repair mechanisms have evolved to combat these hazardous effects. The base excision repair (BER) pathway repairs two of the most common methylated DNA bases, namely 3-methyladenine (3MeA) and 7-methylguanine (7MeG) [1]. BER of these lesions is initiated by the Aag glycosylase through cleavage of the N-glycosyl bond, producing an abasic (AP) site. The apurinic/apyrimidinic endonuclease 1 (Ape1) then cleaves the phosphodiester backbone at the AP site, generating a single-strand break (SSB) with 3’-OH and 5’-deoxyribosephosphate (5’-dRP) ends. DNA polymerase β (Polβ) removes the 5’-dRP and inserts DNA nucleotides to fill the gap. Finally, the SSB is sealed by Ligase I or Xrcc1/Ligase IIIα, completing repair.

BER progression is thought to be tightly coordinated since, if left unrepaired, many of the BER intermediates are toxic. AP sites and SSBs inhibit transcription and replication, potentially generating DNA double-strand breaks (DSBs) [2, 3]. Although translesion polymerases can replicate past AP sites, this often produces point mutations [4, 5, 6, 7]. SSBs are rendered even more toxic during BER if the 5’-dRP termini is not removed by the lyase activity of Polβ [8]. Strikingly, *Polβ* null cells are only methylation sensitive when Aag is present to initiate BER, and suppression of sensitivity only requires expression of Polβ’s dRP lyase domain [8, 9]l. Thus, DNA repair through BER can be hazardous to a cell if enzymatic imbalances exist in the pathway.

BER generated SSBs are quickly bound by Parp1 [10]; upon binding, Parp1 catalyzes the addition of poly-ADP ribose (PAR) polymers to itself and other target proteins [11]. Though Parp1 is not required for accurate completion of BER, activation of Parp1 at SSBs helps recruit the scaffold protein Xrcc1 to stimulate the completion of DNA repair [12, 13, 14, 15]. However, hyperactivation of Parp1 by excessive levels of SSBs can cause cell death in some cell types. Parp1-dependent toxicity is attributed in part to bioenergetic failure due to the rapid loss of cytosolic NAD^+^, which inhibits ATP production. Parp1 can also cause cell death through an independent mechanism wherein PAR polymers translocate to mitochondria, inducing mitochondrial release and nuclear translocation of apoptosis-inducing factor (AIF) [16, 17, 18]. Recently, PAR polymers were found to translocate to mitochondria where they bind and inhibit hexokinase (HK), the initiating enzyme of glycolysis, providing yet another potential mechanism for mediating cell death [19, 20].

Our previous work has demonstrated that MMS-treated mice exhibit cerebellar neurodegeneration and motor function impairment in an Aag-dependent manner [21]; whereas *Aag*^-/-^ mice are strikingly resistant to neurodegeneration, WT mice are sensitive, and *mAagTg* mice with increased expression of *Aag* are hypersensitive. Genetic disruption [21] and pharmacological inhibition (Alocca, et al., in preparation) of Parp1 similarly rescues mice from cerebellar neurodegeneration after alkylation treatment, even in *mAagTg* mice. To further characterize the molecular mechanisms of CGN sensitivity to MMS, we optimized a method for the isolation and culture of primary cerebellar granule neurons (CGNs) from post-natal mouse pups. Here we show that MMS-induced sensitivity of *ex vivo* CGN cultures accurately recapitulates the Aag- and Parp-dependent *in vivo* phenotypes, thus providing a tractable system for the study of cell death pathways. CGN sensitivity to MMS cannot be explained through traditional mechanisms of Parp-mediated cell death, such as NAD^+^ loss, glycolytic inhibition, and AIF translocation. Our results instead suggest an as yet undefined role for Parp1 in a novel pathway for DNA damage-induced cell death in mouse CGNs.

## Materials and methods

### Materials and antibodies

The following materials were purchased from Sigma-Aldrich: Methyl methanesulfonate (Cat. #129925), Zvad-fmk (Cat. #V116), Cyclosporin A (Cat. #30024), BAPTA-AM (Cat. #A1076), β-Nicotinamide adenine dinucleotide hydrate (NAD+, Cat. #N7004), and necrostatin-1 (Cat. #N9037). Sodium pyruvate was purchased from Invitrogen. Parp inhibitors Veliparib and Olaparib were purchased from Selleck Chem. All cell culture components were purchased from Invitrogen unless otherwise noted.

The following antibodies were used in this study: GFAP (AbCam, rabbit polyclonal, ab7779-500, 1:500), Map2 (AbCam, chicken polyclonal, ab5392, 1:20,000), Tuj1 (AbCam, mouse monoclonal, ab7751, 1:500), PAR (Trevigen, mouse monoclonal, 4335-MC-100, 1:2,000), AIF (Cell Signaling Tech., rabbit monoclonal, 5318, 1:500), and CoxIV (Cell Signaling Tech., mouse monoclonal, 11967, 1:200).

### Isolation of cerebellar granule neurons

All methods were reviewed and approved by the Massachusetts Institute of Technology (MIT) Committee on Animal Care (CAC). Animals were euthanized by CO_2_ inhalation followed by cervical dislocation. Cerebellar granule neurons were isolated using an optimized method described elsewhere [22]. Briefly, neurons were isolated from WT, *Aag*^-/-^, and *mAagTg* mouse pups at post-natal day 5–8. Each cerebellum was isolated and plated separately in a genotype blind approach, representing distinct biological replicates. Cerebella were carefully isolated under a dissecting microscope in HBSS-glucose (6 g/L) and the meninges and choroid plexus were removed. The cerebella were dissociated with papain & DNAse (Worthington Biochemical) for 30–45 minutes at 37°C, after which the tissue was further dissociated by trituration with a P1000 pipet. Cells were spun down, resuspended in Earle’s Balanced Salt Solution (EBSS, Worthington), carefully pipetted atop albumin-ovomucoid inhibitor solution (Worthington Biochemical) and centrifuged for 6 minutes at 70x g. Cells were then resuspended in neuronal media containing 10% FBA and run through a 70 μm filter to remove large cells and clumps. Remaining cells were pre-plated on poly-d-lysine (PDL) coated 12-well plates (100 μg/mL) for 2 consecutive 30-minute incubations at 37°C to remove non-neuronal cells, which adhere readily in the presence of FBS. Cells were collected, counted, and diluted to 1 x 10^6^ cell/mL in complete neuronal media (B27/Neurobasal plus 1x L-Glutamine, 1x penicillin/streptomycin, and 250 μM KCl). CGNs were plated on PDL coated dishes (500 μg/mL) at 3,000 cells/mm^2^. For sensitivity assays, cells were plated on optical bottom 96-well plates (Costar 3603) and for immunocytochemistry neurons were plated on glass bottom dishes (Invitrosci). All PDL coated plates were incubated for 2 hours at room temperature. They were then washed twice with water and allowed to dry completely before use.

### Treatment of neurons

Neurons were treated 6–8 days post-plating in neuronal media without B27 for 1 hour at 37°C. Cells were pre-treated with Parp inhibitors (Veliparib/Olaparib) for 1 hour before MMS treatment and Parp inhibitors were included in the media during and after MMS treatment. Neurons were similarly pre-treated with zVad-fmk (100 μM, 3 hours, left in media during treatment), cyclosporine A (50 μM, 1 hour pretreatment and not left in media post-treatment), necrostatin-1 (50 uM, left in media post-treatment), BAPTA (20 μM, 1 hour pretreatment only), or NAD^+^ (various doses, 1 hour pretreatment and present during and after MMS treatment) in complete media at 37°C.

### Determination of cell death

Cell survival was assessed 24 hours post-drug treatment by staining with Calcein-AM (Invitrogen, C3099, 1 μM) and propidium iodide (Invitrogen, 20 μM) for 30 minutes at 37°C. 96-well plates (Costar 3603) were imaged with the ArrayScan XTI High Content Platform (Life Technologies) and the included software algorithm was optimized to identify and count live and dead cells from the green and red fluorescent channels, respectively. Data presented is from biological replicates, where each data point is the average of 2–4 wells, with each well imaged 7 times.

### Mitochondrial permeabilization measurements

Cells were treated with MMS for 1 hour in serum-free media and JC-1 (3 μg/mL) was added at different times post-treatment. Mitochondria were stained for 40 minutes at 37°C, stain was removed and fluorescence was immediately measured with a fluorescent plate reader (VWR SpectraMax ^®^ M3 Multi-Mode Microplate Reader). Mitochondrial decoupler FCCP (carbonyl cyanide 4-(trifluoromethoxy) phenylhydrazone; Sigma C2920; 10 μM) was added during JC-1 incubation and used as a positive control for mitochondrial depolarization.

### Immunofluorescence

Neurons were plated in 24- or 96-well glass bottom plates (Invitrosci) and fixed with 4% paraformaldehyde for 15–30 minutes. Cells were then permeabilized with 0.1% Triton-X in CSK buffer (100 mM NaCl, 300 mM sucrose, 10 mM PIPES, 3 mM MgCl_2_, 1 mM EGTA) for 20 minutes, blocked with 4% bovine serum albumin (BSA) in PBS for 1 hour at 37°C, and stained overnight at 4°C with appropriate dilutions of antibodies in 4% BSA/PBS. Secondary antibodies were added for 2 hours and neurons were stained with Hoechst for 15 minutes. Images were taken with a Hamamatsu ORCA-R^2^ CCD Camera on a Zeiss Axio Observer.Z1 Microscope.

Neurons stained with anti-PAR were imaged and quantified with the ArrayScan XTI High Content Platform (Life Technologies). 7 images were taken per well at 10X magnification and the included software algorithm was used to quantify the total nuclear fluorescence using Hoechst staining as a nuclear mask.

### *Aag* mRNA fluorescence *In situ* hybridization (FISH)

Neurons were plated on PDL-coated 28-mm glass bottom dishes (Invitrosci) and fixed 7 days later with 4% paraformaldehyde for 20 minutes at room temperature. Cells were permeabilized with cold 70% ethanol overnight at 4°C and washed the following morning for 5 minutes with wash buffer (25% formamide, 2X SSC). Neurons were stained with 40 20-nucleotide-long fluorescently labeled (Alexa-647) oligonucleotides designed against the *Aag* transcript overnight at 37°C in a heavily humidified chamber in hybridization buffer (100 mg/mL dextran sulfate, Sigma, D8906: 0.5 mg/mL E.coli tRNA, Sigma, R4251: 0.5 mg/mL ssDNA, Sigma D9156: 1 mg/mL Ultapure BSA, Ambion, AM2616: 10 mM VRC, New England Biolabs, S1402S: 25% formamide, Ambion, AM9342: 2X SSC, Ambion, AM9763). Finally, cells were stained with DAPI (2 μg/mL) in wash buffer for 30 minutes at 37°C before imaging. All the previous steps were done in nuclease-free solutions to avoid RNA degradation. We occasionally see faint transcripts in *Aag*^-/-^ cells due to initiation of endogenous transcription before hitting the inserted cassette that disrupts the gene.

Images were taken with a Hamamatsu ORCA-R^2^ CCD Camera on a Zeiss Axio Observer.Z1 Microscope. 21 Z-stack images were taken, each 0.3 μm away from the previous. Images were compiled and foci per cell were counted with a MATLAB algorithm adapted from previously published methods [23].

### Host cell reactivation assay

Plasmids were engineered to contain a site-specific hypoxanthine (Hx) lesion in the fluorophore codon of the gene encoding GFP as described in [24]. In the absence of Aag, Hx causes transcriptional mutagenesis and miscodes for a C, leading to the fluorescent variant of the protein. If Aag-initiated BER repairs Hx, the appropriate U is incorporated and no fluorescence is observed. To test for the presence of Hx in our plasmids, 150 ng of the plasmids were incubated with 10 units ApaLI (NEB) in 1X Cutsmart buffer for 1 hour at 37°C, followed by 20 min at 65°C for heat-inactivation. Products were run on a 1% agarose gel for visualization.

Neurons were plated on 96 well plates and plasmids were transfected using Lipofectamine 3000 (Invitrogen) 6–7 days post-isolation according to manufacturer’s instructions using 2.5 μg of total plasmid DNA. 5 wells per biological replicate were transfected with either damaged or undamaged plasmids. The plate was immediately placed into the IncuCyte Zoom (Essen Bioscience) live cell automated fluorescent imager. 4 images per well were taken every hour (phase contrast and red/green fluorescent filters). Included software was used to identify cells based on phase contrast and quantify integrated cellular fluorescence.

### Gene expression analysis

Neurons were harvested for RNA isolation and gene expression analysis 1 week after plating in a 6 cm plate. Neurons were washed twice with HBSS and harvested by gentle cell scraping. Cells were resuspended in TRIzol (Invitrogen) and RNA was isolated with the RNeasy Mini Kit (Qiagen). 1 μg of RNA was converted to cDNA with Omniscript reverse transcriptase following manufacturer’s instructions (Qiagen).

cDNA was measured using a ABI Fast Cycler and the following Taqman probes (Invitrogen): *Pol β* (Mm00448234_m1), *Ape1* (Mm01319526_g1), *Xrcc1* (Mm00494222_m1), *Parp1* (Mm01321084), *Aag* (Mm00447872_m1), *Lig3* (Mm00521933_m1) and *Map2* (Mm99999915_g1). Map2 was used as the normalization control.

## Results

### Expression levels of Aag in primary cerebellar granule neurons determines MMS sensitivity

**Fig 1:**
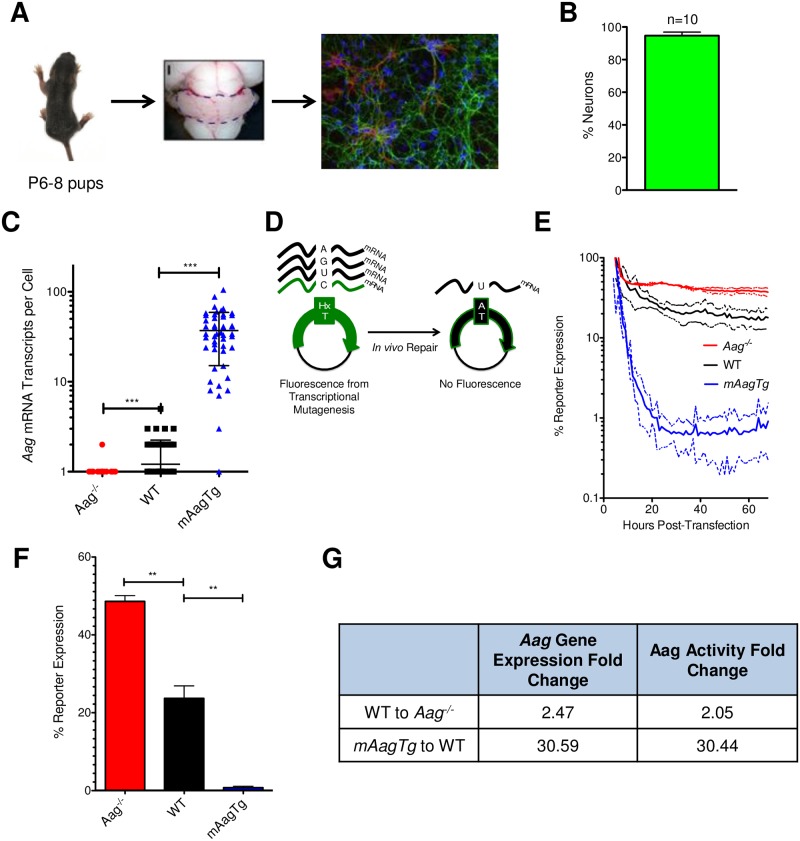
Isolation and characterization of *Aag*^-/-^ cerebellar granule neurons. (A) Neurons were isolated from mouse cerebella at post-natal day 5–8. Representative immunocytochemical image depicts neurons (Map2, green) and astrocytes (GFAP, red). Nuclei are shown in blue. (B) Map2+ neurons were quantified from images from 10 pups isolated on different days. Error bars represent standard deviation from the mean. (C) *Aag* transcripts were counted per cell and are plotted by genotype, where each data point represents one cell. Errors bars denote standard deviation from the mean, n ≥ 50 cells, *** p<0.001 using Student’s standard two-tailed T-test. (D) Cartoon representation of the host-cell reactivation method to measure Aag glycosylase activity in cells. (E) Glycosylase activity over time by cellular fluorescent output from host cell reactivation assay. Solid lines denote mean while dashed lines indicate standard deviation from the mean. (F) WT, *Aag*^-/-^ and *mAagTg* neurons have significantly different Aag glycosylase activity at 24 hours post-transfection. Errors bars denote standard deviation from the mean. WT n = 3, *mAagTg* n = 2, *Aag*^-/-^ n = 2. ** p<0.01 using Student’s standard two-tailed T-test). (G) Fold changes between WT, *Aag*^-/-^ and *mAagTg* Aag expression and glycosylase activity.

Prior to studying the molecular mechanisms of MMS-induced cell death *ex vivo*, we had to verify that the neuronal responses to alkylation treatment were cell-intrinsic properties, i.e., not dependent on the complex environment of the cerebellum. To this end, we optimized a method for the isolation of cerebellar granule neurons (CGNs) from mouse pups to study the effects of alkylation treatment in individual cells cultured *ex vivo* [22] (Fig 1A). Cerebella were isolated from WT, *Aag*^-/-^, and *mAagTg* pups and immunocytochemical analysis demonstrated that the resulting primary cultures were ~95% mature Map2 expressing neurons (Fig 1A and 1B).

We determined the difference in *Aag* expression and activity between the three genotypes. mRNA fluorescence *in situ* hybridization (FISH) was employed to determine the number of *Aag* transcripts in individual neurons. *Aag* expression was significantly different between the three genotypes, with an average transcript number per cell of 0.49, 1.21, and 37.04 in *Aag*^-/-^, WT, and *mAagTg* neurons, respectively (Fig 1C). Given the near complete MMS resistance in *Aag*^-/-^ CGNs *in vivo* relative to the sensitivity seen in WT CGNs, we were surprised to find that the difference in Aag expression between WT and *Aag*^-/-^ was relatively small (~2.5-fold), but nonetheless statistically significant (p < 0.0001, Fig 1C). Most striking, however, was the large increase and variability in *Aag* expression in the *mAagTg* neurons, with between ~10–100 *Aag* transcripts per neuron (Fig 1C).

We measured *Aag* glycosylase activity using a host-cell reactivation (HCR) method based upon fluorescent protein output [24]. Plasmids with site-specific hypoxanthine lesions (known to be repaired exclusively by Aag-initiated BER) were transfected into primary neurons and cellular fluorescence was monitored over time with the IncuCyte Zoom Live Cell imager (Essen BioScience). In this HCR assay, reporter expression is inversely correlated with Aag activity because fluorescent protein is only generated by transcriptional mutagenesis past the hypoxanthine lesion; increased Aag activity, and thus increased hypoxanthine removal, results in decreased expression of the fluorescent protein (Fig 1D). We monitored Aag activity over time and found that after an initial stabilization period of roughly 20 hours, the percent reporter expression stabilized for all genotypes (Fig 1E). There were statistically significant differences in Aag activity between the 3 genotypes at the 24-hour timepoint after transfection (Fig 1F). Moreover, the fold changes in mRNA *Aag* expression between genotypes correlated well with fold changes in Aag activity (Fig 1G).

**Fig 2:**
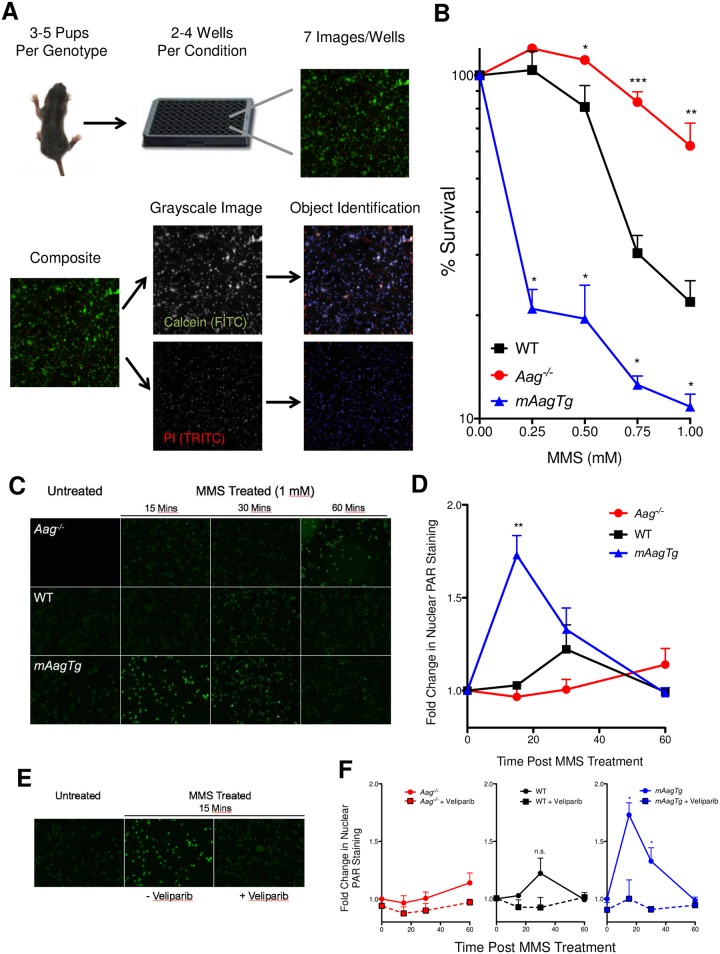
CGN sensitivity to MMS is dependent on Aag and involves activation of Parp. (A) Overview of CGN toxicity assay based on high content imaging. (B) Sensitivity to MMS treatment *ex vivo* is dependent on Aag. Errors bars denote standard error from the mean, *Aag*^-/-^: n = 6, WT: n = 5, *mAagTg*: n = 3. * p<0.05, ** p<0.01, *** p<0.0001 using Student’s standard two-tailed T-test comparing a single dose to WT. (C) PAR formation (green) was visualized in *Aag*^-/-^, WT, and *mAagTg* neurons 0, 15, 30, or 60 minutes after the addition of MMS (1 mM) by immunocytochemical staining. (D) Fold changes in nuclear PAR staining was quantified and reflects qualitative trends in (C). Errors bars denote standard error from the mean. *Aag*^-/-^: n = 4, WT: n = 3, *mAagTg*: n = 2. **p<0.01 using Student’s standard two-tailed T-test comparing nuclear fluorescence at a particular timepoint compared to untreated cells. (E) Representative images of PAR formation in *mAagTg* neurons either left untreated, or 15 minutes after MMS treatment (1 mM) with or without the addition of Veliparib (5 μM). (F) Quantification of changes in nuclear PAR formation in neurons with or without Veliparib pretreatment. Errors bars denote standard error from the mean. *Aag*^-/-^: n = 4, WT: n = 3, *mAagTg*: n = 2. * p<0.05 using Student’s standard two-tailed T-test comparing nuclear fluorescence with or without Veliparib at a particular time post MMS treatment.

To assess sensitivity to DNA damaging agents, we established a method to assess neuron sensitivity to MMS based on staining with the fluorescent dyes Calcein and propidium iodide (PI) that stain live and dead neurons, respectively. Fluorescent imaging and live/dead cell identification and counting was conducted with the ArrayScan XTI High Content platform (Thermo Scientific) (Fig 2A). Using this methodology, we found that *Aag*^-/-^ neurons are significantly resistant to MMS-induced death compared to WT. Moreover, increased expression of *Aag* in *mAagTg* neurons renders them extremely sensitive to MMS (Fig 2B). These results establish that CGN sensitivity to MMS is an intrinsic cellular phenotype that can be studied *ex vivo*.

### Aag-dependent CGN sensitivity to MMS is mediated through poly (ADP-Ribose) polymerase activity

Parp1 activity is known to exacerbate the severity of many neurodegenerative diseases, including stroke and Alzheimer’s disease [25, 26, 27]. Indeed, our lab has previously demonstrated that genetic loss of Parp1 rescues cerebellar and retinal neurodegeneration post-MMS treatment in WT and *mAagTg* mice [21]. Upon binding to single-strand DNA breaks, Parp1 catalyzes the addition of PAR polymers onto itself and other protein targets; the PAR polymers are later degraded by poly (ADP-ribose) glycohydrolase (Parg). Since SSBs are transiently created during BER, we anticipated that Aag activity should correlate with the magnitude and dynamics of PAR formation after MMS treatment; for example, increased Aag activity should lead to increased production of AP sites that when converted to SSBs will activate Parp. We assessed nuclear PAR formation following the addition of MMS by immunocytochemical staining and saw a large and statistically significant increase in nuclear PAR 15 minutes post-MMS addition in *mAagTg* neurons, which subsequently fell to basal levels by 60 minutes post-treatment (Fig 2C and 2D). There was a slight but statistically non-significant increase in PAR formation in WT neurons 30 minutes post treatment (p = 0.168), while PAR formation in *Aag*^-/-^ neurons remained near basal levels over the 60 minute timeframe. Addition of the PARP inhibitor Veliparib completely suppressed PAR formation after treatment with MMS in WT and *mAagTg* CGNs (Fig 2E and 2F). Such Aag dependent increases in PAR formation after MMS treatment support the idea that imbalanced BER due to variable Aag activity is the source of MMS-induced CGN toxicity.

**Fig 3:**
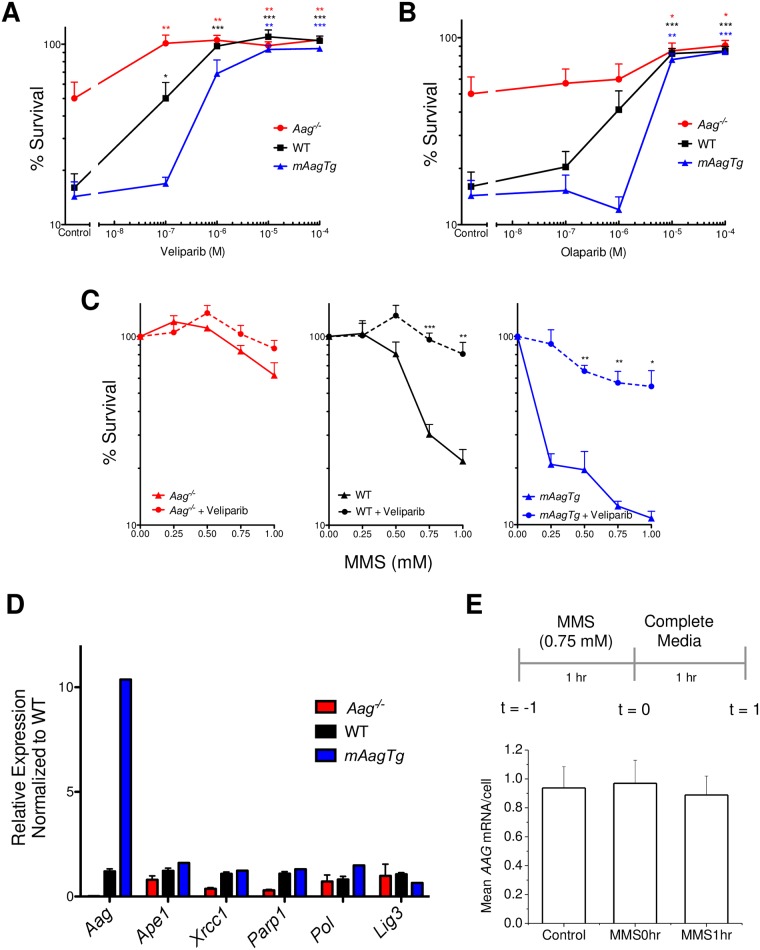
Parp inhibition rescues CGN sensitivity to MMS. *Aag*^-/-^, WT, and *mAagTg* neurons are rescued from MMS toxicity (1 mM) after pretreatment with Parp inhibitor Veliparib (A) or Olaparib (B). Errors bars denote standard error from the mean, *Aag*^-/-^: n = 6, WT: n = 5, *mAagTg*: n = 3. * p<0.05, ** p<0.01, *** p<0.0001 using Student’s standard two-tailed T-test compared to neurons of the same genotype treated with 1 mM MMS (control). (C) Parp inhibitor Veliparib rescues cell sensitivity at all doses of MMS in WT and *mAagTg* neurons. Errors bars denote standard error from the mean, *Aag*^-/-^: n = 6, WT: n = 5, *mAagTg*: n = 3. * p<0.05, ** p<0.01, *** p<0.0001 using Student’s standard two-tailed T-test comparing sensitivity with or without Veliparib for a genotype at a particular dose of MMS. (D) Expression of BER genes in CGNs. Errors bars denote standard deviation from the mean, *Aag*^-/-^ n = 4, WT n = 5, *mAagTg* n = 1. (E) Aag expression was assessed either before, immediately after, or an hour after MMS treatment using *Aag* mRNA FISH in WT CGNs. Errors bars denote standard deviation from the mean.

We next tested the ability of Parp inhibitors to rescue CGN mediated MMS-induced neurotoxicity, using two clinically relevant Parp inhibitors, Veliparib and Olaparib. Addition of increasing amounts of either Parp inhibitor rescued MMS sensitivity to untreated control levels (Fig 3A and 3B). Similarly, Parp inhibitors conferred significant rescue of WT and *mAagTg* neurons as the dose of MMS was increased, indicating that Parp1 activity mediates alkylation induced toxicity at both low and high levels of DNA damage (Fig 3C). Surprisingly, Parp inhibitors offered slight rescue even in *Aag*^-/-^ neurons at 1 mM MMS, though not to a statistically significant level (Fig 3C, p = 0.108). Damaged DNA bases such as 7MeG or 3MeA spontaneously depurinate [28, 29], initiating BER independent of Aag activity; such BER activity could lead to Parp1 activation. Parp inhibitors may therefore facilitate MMS rescue even in the absence of Aag. We conclude that Aag-initiated CGN death post-MMS treatment is facilitated through Parp1 activity *ex vivo*, further corroborating our *in vivo* results and indicating that the CGN response to MMS is cell-intrinsic.

One might argue that changes in Aag expression cause changes in the expression of downstream BER enzymes and that these changes contribute to changes in MMS sensitivity. We therefore measured the expression of a panel of BER genes in all three genotypes to exclude this possibility. As shown in Fig 3E, there were large statistically significant differences in the expression of *Aag* between *Aag*^-/-^, WT, and *mAagTg* CGNs. In contrast, there were no significant differences in the expression of *Ape1*, *Xrcc1*, *Pol β*, *Parp1*, and *Lig3* between *Aag*^-/-^, WT, and *mAagTg* CGNs (Fig 3D). Moreover, *Aag* expression did not change after MMS treatment, indicating that the basal *Aag* expression and protein activity levels are sufficient to confer significant differences in cell sensitivity to MMS (Fig 3E).

### Potential modulators of neuron sensitivity to MMS

**Fig 4:**
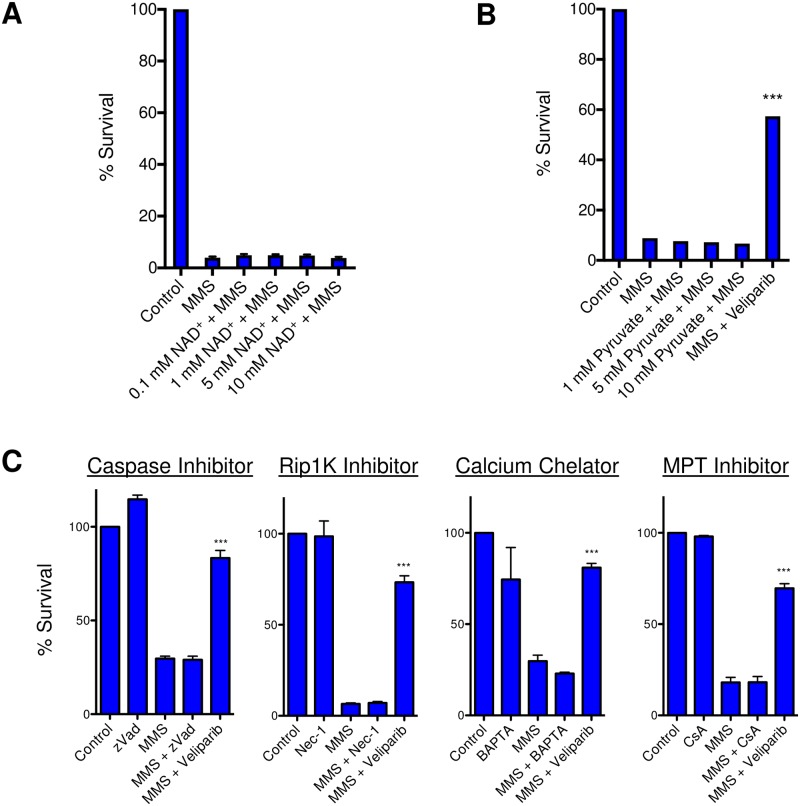
mAagTg CGN sensitivity to MMS is not dependent on NAD+, pyruvate, caspases, Rip1K, calcium fluxes, or MPT. (A) NAD^+^ pre-treatment did not rescue *mAagTg* neuron sensitivity to MMS. Errors bars denote standard deviation from the mean. *mAagTg* n = 3. (B) Pyruvate did not rescue *mAagTg* neuron sensitivity to MMS. Errors bars denote standard deviation from the mean. *mAagTg* n = 1. *** p<0.001 using Student’s standard two-tailed T-test comparing MMS and MMS + Veliparib. (C) Primary *mAagTg* CGN sensitivity to MMS is not dependent on caspase activation, Rip1 kinase activity, calcium fluxes, or mitochondrial permeability transition (MPT). Inhibitors used include zVad-fmk (zVad), n = 6, Necrostatin-1 (Nec-1), n = 3, BAPTA-AM, n = 3, and cyclosporin A (CsA), n = 3. Errors bars denote standard error from the mean. *** p<0.001 using Student’s standard two-tailed T-test comparing MMS to MMS+Veliparib.

At moderate levels of DNA damage, PAR formation is thought to facilitate DNA repair by recruiting necessary BER proteins; however, Parp hyperactivation in response to excessive DNA damage is hypothesized to cause cell death due to excessive consumption of cellular NAD^+^, leading to bioenergetic failure [11]. Some have reported that addition of exogenous NAD^+^ is sufficient to rescue alkylation-induced toxicity [19, 30]. However, we found that the addition of NAD^+^ was unable to provide any rescue to *mAagTg* CGNs after MMS (Fig 4A). To further validate these results, we tested whether the addition of pyruvate could rescue PARP-mediated CGN toxicity by bypassing any inhibition of glycolysis that may be caused by cellular loss of NAD^+^, thus directly supplementing the TCA cycle and avoiding bioenergetic failure [19, 30]. Like NAD^+^, pyruvate was unable to rescue MMS induced toxicity (Fig 4B). We infer that in CGNs Parp mediates Aag-dependent MMS-induced cell death through an alternative pathway that does not involve NAD^+^ depletion.

Cell death caused by Parp hyperactivation is considered a form of programmed necrosis (necroptosis) and is often, though not always, executed independently of pro-apoptotic caspases [31, 32]. Indeed, we found that addition of the pan-caspase inhibitor zVad-fmk did not affect *mAagTg* CGN sensitivity to MMS (Fig 4C). Programmed necrosis can be initiated by numerous different stimuli, resulting in a variety of cellular outcomes that ultimately conclude in what appears as necrotic cell death [31, 33]. Canonical necroptosis initiated by the extra cellular ligand tumor necrosis factor alpha (TNFα) is mediated through the receptor-interacting protein kinase 1 (Ripk1) and Ripk3. Necrostatin-1 (Nec-1) is a potent small molecule kinase inhibitor of Ripk1 that was identified by its ability to inhibit TNF induced necrotic cell death [34]. Pre-incubation of neurons with Nec-1 was not able to rescue *mAagTg* sensitivity to MMS (Fig 4C). This is not entirely surprisingly given that Ripk1 and Parp1 activities have been shown to represent two distinct cell death pathways [35, 36].

Finally, we examined other known modulators of programmed necrosis. Fluctuations in calcium concentrations can cause neuronal death through the activation of the calpains, calcium-dependent cysteine proteases that can cleave mitochondrial apoptosis inducing factor (AIF) leading to its translocation to the nucleus where it can help execute cell death [37, 38, 39]. However, in our system, there was no effect on cell death when calcium was chelated to prevent fluxes (Fig 4C). We also queried whether mitochondrial permeability transition (MPT) was occurring through opening of the MPT pore (MPTP) since MPT is known to play an important role in neuronal necrosis in response to excitotoxicity and ischemia-reperfusion injury [40, 41, 42]. The MPTP sits within the inner mitochondrial membrane and upon opening, creates a pore connecting the cytosol and inner mitochondria that is permeable to all molecules under 1.5 kDa. Inhibition of MPT with cyclosporine A (CsA), however, did not rescue the sensitivity of CGNs to MMS (Fig 4C).

**Fig 5:**
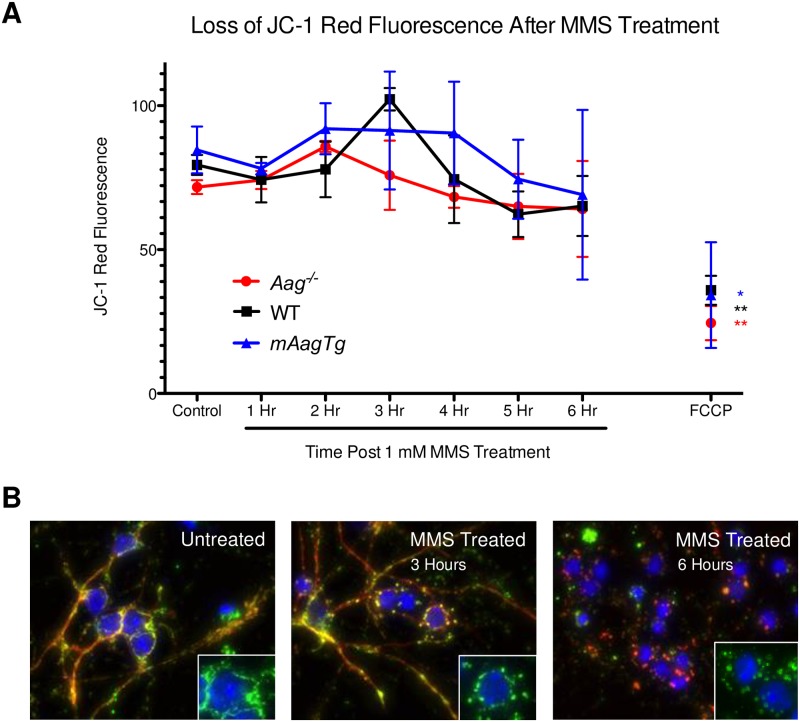
No loss in mitochondrial permeability or translocation of AIF in neurons after MMS treatment. (A) There is no loss of mitochondrial permeability in *Aag*^-/-^, WT, or *mAagTg* neurons 1–7 hours after MMS treatment. Errors bars denote standard deviation from the mean. *Aag*^-/-^ n = 2, WT n = 3, *mAagTg* n = 2. ** p<0.01 using Student’s standard two-tailed T-test comparing FCCP to control. * p<0.05 using Student’s standard one-tailed T-test comparing FCCP to control. (B) WT CGNs treated with MMS do not show evidence of AIF (red) translocation from the mitochondria (green; CoxIV) to the nucleus (blue; Hoechst).

Even though MPT did not occur, this does not preclude the possibility that mitochondria were becoming depolarized through other mechanisms. We monitored mitochondrial permeability 1–6 hours post-MMS treatment with the fluorescent mitochondrial polarization marker JC-1. We found no evidence of mitochondrial depolarization within this timeframe. The positive control, mitochondrial decoupler FCCP demonstrated significant loss of mitochondrial polarization, showing that depolarization is possible in this cell type (Fig 5A).

Translocation of apoptosis inducing factor (AIF) from the mitochondria to the nucleus is often seen in Parp1-mediated alkylation sensitivity and is believed to stimulate nuclear DNA fragmentation [17, 18]. However, we found no evidence of AIF translocation in WT neurons either 3 or 6 hours post-MMS treatment (Fig 5B). Surprisingly, we did observe the development of an interesting mitochondrial structure at these timepoints, which have been termed ‘donut’ or ‘blob’ mitochondria. These abnormal mitochondrial shapes have been strongly associated with mitochondrial stress in many cell types, including neurons, after ischemia-reperfusion damage [43, 44]. Taken together, these results indicate that mouse CGNs undergo a novel Parp1-dependent cell death mechanism after DNA alkylation damage.

## Discussion

Initiation of BER by the Aag DNA glycosylase after MMS treatment induced primary CGN cell death that is mediated through Parp activation. Pharmacological inhibition of Parp activity was sufficient to rescue neuronal sensitivity by preventing PAR polymer formation as did deletion of the *Aag* gene. We used previously published reports of Parp1 mediated toxicity to DNA damage as a guide in our search for the downstream molecular mechanisms of cell death in CGNs. Intriguingly, murine CGN sensitivity to MMS *ex vivo* could not be explained by the action of previously established mediators of Parp1-dependent cell death, and thus represents a novel cell death pathway.

Pharmacological inhibition of Parp rescued MMS-induced neuron sensitivity, similar to that seen *in vivo* upon genetic deletion of Parp1 [21]. However, it is important to note that Parp deficiency and Parp inhibition do not always cause the same phenotypes, as in this case. Parp inhibitors, including Veliparib and Olaparib, function by competing with NAD^+^ for the NAD^+^ binding site within the enzyme. Inhibition of Parp1 activation upon binding to single-strand breaks prevents SSB-induced auto-PARylation and release of Parp1 from DNA; a Parp1-DNA complex is thus created, which in some cases can become covalently linked [45, 46]. Such protein-DNA crosslinks can themselves be cytotoxic by impeding the transcription and replication machinery, resulting in single- and double-strand breaks [47, 48, 49]. Thus while Parp inhibition and Parp1 knockout both result in suppression of cellular signaling via Parp, pharmacological inhibition of Parp has the capacity to increase total levels of DNA damage post methylation treatment. Olaparib has been shown to be a more potent creator of Parp1-DNA complexes than Veliparib [50], yet we found no difference in the capacity of either inhibitor to rescue MMS sensitivity (Fig 2C and 2D). Furthermore, double-strand break formation after Parp inhibition is dependent on cells being in S-phase of the cell cycle [48]. Given the post-replicative nature of mature neurons, it is perhaps not surprising that Parp inhibition did not exacerbate sensitivity to alkylating agents but suggests that Parp inhibitors may be a viable option to minimize neuronal loss during alkylation chemotherapy.

Programmed necrosis is initiated by numerous stimuli, causing cell death through a variety of potentially overlapping mediator steps in addition to distinct cell death pathways including necroptosis, ferroptosis, parthanatos, and pyroptosis [31, 33]. Parp1 hyperactivation is one such well-established mediator of programmed necrosis after excessive DNA damage, catalyzing the formation of PAR polymers and consuming cytosolic NAD^+^ in the process [51]. It has been postulated that this reduction in NAD^+^, and subsequent loss of ATP, is sufficient to cause cell death [30]. Yet, we found that the addition of NAD^+^ did not rescue Aag- and Parp1-dependent neuronal sensitivity. However, recent publications have demonstrated that this sequence of events may not always be true because ATP depletion can precede NAD^+^ loss after alkylation treatment [19, 20]. Moreover, PAR polymers were shown to translocate to the mitochondria and bind directly to hexokinase (HK), the initiating enzyme of glycolysis, thus inhibiting its activity [19, 20]. This glycolytic inhibition was independent of NAD^+^ loss and glycolysis could be restored through the addition of tricarboxylic acid (TCA) cycle substrates pyruvate or glutamine [19]. However, we found no rescue in neuronal viability after MMS treatment upon the addition of pyruvate, again suggesting that Parp is mediating cell death through a novel mechanism independent of glycolytic inhibition but potentially involving the mitochondria.

PAR translocation to mitochondria has also been shown to induce mitochondrial release of AIF [17, 18]. AIF translocates to the nucleus where it initiates chromatinolysis through interaction with histone H2AX and cyclophilin A [52]. Yet, there was no evidence of AIF nuclear translocation after MMS treatment in our cultured neurons (Fig 5B). However, it remains possible that we did not choose the relevant timepoint and therefore failed to capture AIF translocation.

It is usually assumed that PAR polymers synthesized in the nucleus cause mitochondrial effects; however, it cannot be ignored that Parp1 itself has been shown to partially localize to the mitochondrial [53, 54]. Mitochondrial Parp1 has been found to interact with the mitochondria specific DNA replication and repair proteins exonuclease G and DNA polymerase γ [55]; additionally, mitochondrial Parp1 was identified in a DNA-binding protein complex containing DNA Ligase III [54]. Despite these interactions, there is inconsistency in the literature about whether Parp1 maintains or promotes mitochondrial genetic instability [54, 55]. PARylation was found on a variety of mitochondrial proteins involved in cellular bioenergetics and homeostasis after oxidative damage [56, 57]. Finally, inhibition of mitochondrial PAR prevented cell death caused by oxidative damage [53]. Taken together, these results indicate that mitochondrial-derived PARylation might be more common than expected and may contribute to Parp-dependent mitochondrial dysfunction.

Inhibition of calcium fluxes via calcium chelation did not alter CGN sensitivity to MMS. Increases in cellular calcium concentration occurring during programmed necrosis are responsible for the activation of calpains, calcium dependent intracellular cysteine proteases, that can cleave AIF and the pro-apoptotic protein Bid to tAIF and tBid, respectively. There are debates in the literature, but under certain circumstances, calpain activation, not PAR translocation, is essential for the release of tAIF from the mitochondria after alkylation treatment [58, 59, 60]. Calcium chelation has also been shown previously to rescue oxidation but not alkylation induced death [61]. Given the variety of results seen in the literature, it is likely that the downstream mediators of programmed necrosis are highly dependent on cell type and treatment conditions.

Though we found no evidence of mitochondrial depolarization after MMS treatment, we did observe the formation of unique ‘donut’ mitochondrial structures. This mitochondrial shape is associated with swelling, alterations in mitochondrial fusion dynamics, increased ROS and overall poor mitochondrial health in individually depolarized mitochondria [43, 44]. ‘Donut’ mitochondria formation has similarly been documented after ischemia/reperfusion, oxidative stress, and glutamate excitotoxicity [44, 62, 63, 64, 65]. Finally, ‘donut’ mitochondria withstand stress better than tubular mitochondria, possibly due to their ability to redistribute and mix matrix components more easily, so their formation may simply be an attempt at survival post-methylation treatment [44]. It is not clear whether these donut mitochondrial structures are causative of cell death or merely correlated. The mitochondrial phosphatase PGAM5 has been identified as an important regulator of programmed necrosis signaling pathways, ultimately causing cell death through mitochondrial fragmentation [66]. Similarly, Huntington’s disease associated neurodegeneration was attenuated by inhibition of mitochondrial fission [67]. Therefore, it is possible that mitochondrial fission/fusion dynamics are a causative mechanism during programmed necrosis in primary CGNs after DNA alkylation damage and warrants further investigation.

Our results demonstrate that *ex vivo* cultures of primary murine CGNs recapitulate *in vivo* Aag- and Parp1- dependent sensitivity to MMS treatment, thus representing an accurate model with which to study molecular mechanisms of cell death. We have shown that the cell death is independent of caspase or Rip1K activity, AIF nuclear translocation and mitochondrial depolarization. Moreover, the addition of exogenous NAD^+^ and pyruvate was unable to rescue Aag-dependent MMS sensitivity. Overall, these results suggest a hitherto unreported pathway for Parp1 dependent programmed necrosis after DNA damage that will require additional investigation to fully elucidate.
